# The effect of novel mutations on the structure and enzymatic activity of unconventional myosins associated with autosomal dominant non-syndromic hearing loss

**DOI:** 10.1098/rsob.140107

**Published:** 2014-07-30

**Authors:** Tae-Jun Kwon, Se-Kyung Oh, Hong-Joon Park, Osamu Sato, Hanka Venselaar, Soo Young Choi, SungHee Kim, Kyu-Yup Lee, Jinwoong Bok, Sang-Heun Lee, Gert Vriend, Mitsuo Ikebe, Un-Kyung Kim, Jae Young Choi

**Affiliations:** 1 School of Life Sciences, KNU Creative BioResearch Group (BK21 plus project), Kyungpook National University, Daegu, South Korea; 2 Department of Otolaryngology, College of Medicine, Kyungpook National University, Daegu, South Korea; 3 Soree Ear Clinic, Seoul, South Korea; 4 Department of Microbiology and Physiological Systems, University of Massachusetts Medical School, Worcester, MA, USA; 5 Centre for Molecular and Biomolecular Informatics, Radboudumc, Nijmegen, The Netherlands; 6 Department of Medicine, University of Pennsylvania, Philadelphia, PA, USA; 7 Department of Otolaryngology, Fatima Hospital, Daegu, South Korea; 8 Department of Anatomy, Yonsei University College of Medicine, Seoul, South Korea; 9 BK21 Project for Medical Science, Yonsei University College of Medicine, Seoul, South Korea; 10 Department of Otorhinolaryngology, Yonsei University College of Medicine, Seoul, South Korea

**Keywords:** myosin, mutation, ATPase, protein structure, gene

## Abstract

Mutations in five unconventional *myosin* genes have been associated with genetic hearing loss (HL). These genes encode the motor proteins myosin IA, IIIA, VI, VIIA and XVA. To date, most mutations in *myosin* genes have been found in the Caucasian population. In addition, only a few functional studies have been performed on the previously reported myosin mutations. We performed screening and functional studies for mutations in the *MYO1A* and *MYO6* genes in Korean cases of autosomal dominant non-syndromic HL. We identified four novel heterozygous mutations in *MYO6*. Three mutations (p.R825X, p.R991X and Q918fsX941) produce a premature truncation of the myosin VI protein. Another mutation, p.R205Q, was associated with diminished actin-activated ATPase activity and actin gliding velocity of myosin VI in an *in vitro* analysis. This finding is consistent with the results of protein modelling studies and corroborates the pathogenicity of this mutation in the *MYO6* gene. One missense variant, p.R544W, was found in the *MYO1A* gene, and *in silico* analysis suggested that this variant has deleterious effects on protein function. This finding is consistent with the results of protein modelling studies and corroborates the pathogenic effect of this mutation in the MYO6 gene.

## 2. Introduction

Myosins comprise a large family of motor proteins in eukaryotic tissues. While originally described in muscle tissue, another form of myosin, called unconventional myosin, has been found in many other tissues [1,2]. Unconventional myosins are associated with a diverse number of functions, including endocytosis, the regulation of ion channels and the movement of vesicles in the cytoplasm [3]. In the inner ear, myosins are expressed in the stereocilia and the cell body of the inner and outer hair cells. They bind actin filaments and hydrolyse adenosine triphosphate (ATP) to generate force and movement as well as to anchor hair cell stereocilia of the inner ear [4]. This arrangement enables the stereocilia to bend to sound waves, thereby opening ion channels, which allows for the transduction of sound (the conversion of sound waves to nerve impulses) [5].

To date, mutations in five different types of unconventional myosins—myosin IA (*MYO1A*), myosin IIIA (*MYO3A*), myosin VI (*MYO6*), myosin VIIA (*MYO7A*) and myosin XVA (*MYO15A*)—have been identified in hereditary hearing loss (HL) [6–13]. Among these, the *MYO1A* (OMIM 601478) and *MYO6* (OMIM 600970) genes have been identified as the cause of autosomal dominant non-syndromic hearing loss (ADNSHL) [10,13]. *MYO1A*, which is also known as the brush border myosin-I gene, is located at the DFNA48 locus on chromosome 12, and thus far, eight different mutations have been identified in Italy in patients with HL [13,14]. The role of *MYO1A* in the inner ear and the impact of *MYO1A* mutations on HL in general, however, remain unclear. *MYO6* is located on chromosome 6 [10,12]. Mutations in the *MYO6* gene cause both ADNSHL (DFNA22) and autosomal recessive non-syndromic hearing loss (DFNB37). Previous studies have reported that myosin VI transports cargo molecules at the base of stereocilia, anchors actin filaments to the membrane of stereocilia by cargo molecules in the inner ear and is a mechanism for HL in myosin VI-null mice [15,16].

To date, most missense mutations among the previously reported mutations in the myosin family have only been described at the genome level. All known missense mutations are important for the protein's structure and function because their residue positions are highly conserved among many species. Therefore, a functional evaluation *in vitro* or *in vivo* is required for an accurate characterization of the mutated proteins.

In this study, we identified *MYO1A* and *MYO6* mutations in patients with ADNSHL in Korea and analysed the molecular reasons for the pathogenic effect of novel mutations using *in silico* and *in vitro* analyses.

## 3. Results

### 3.1. Mutation screening of *MYO6* and *MYO1A*

**Figure 1.**
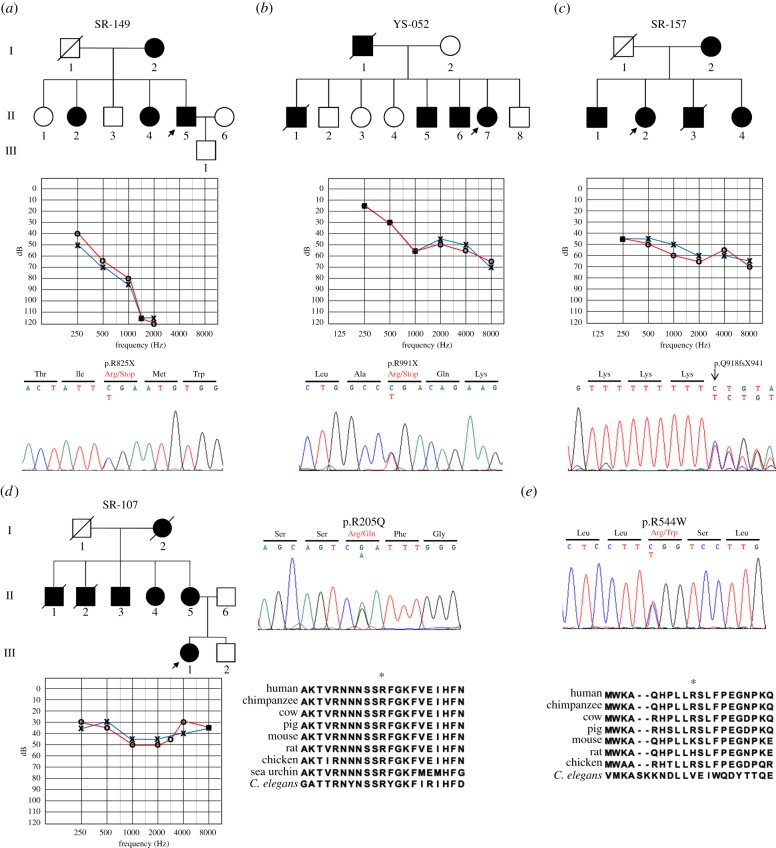
Pedigree and genetic information for patients with novel mutations in the *MYO6* and *MYO1A* genes. The lineages (*a*) SR-149, (*b*) YS-052, (*c*) SR-157 and (*d*) SR-107 exhibit autosomal dominant inheritance patterns. Two nonsense mutations, one frameshift mutation and one missense mutation were identified in these families by direct sequencing (*c*: reverse sequence). The arginine at amino acid position 205 of myosin VI is highly conserved among several species. (*e*) DNA sequence shows the p.R544W variant in the *MYO1A* gene. A comparison of amino acid sequences of myosin IA between several species. The asterisk indicates the mutation site. Squares, males; circles, females; slashes, deceased; shaded, affected. The arrows indicate the proband of each family.

We sequenced the *MYO6* and *MYO1A* genes in 53 unrelated Korean ADNSHL patients. Table 1 shows the four novel heterozygous mutations that were identified in the coding regions of the *MYO6* gene. Two nonsense mutations, p.R825X and p.R991X, were identified in exons 24 and 28 of *MYO6*, respectively. The p.R825X mutation was detected in patient II-5 of the SR-149 family (figure 1*a*). This mutation is a C to T transition at nucleotide position 2473 (c.2473 C > T), which results in a stop codon in the IQ domain of the neck region. The p.R991X mutation was identified in the YS-052 family (figure 1*b*). The single-nucleotide change of C to T at nucleotide position 2971 (c.2971 C > T) introduced a stop codon corresponding to amino acid position 991. This stop codon causes the protein to lose part of the globular domain of the tail region. Patient II-2 of the SR-157 family had a single-nucleotide insertion at position 2752 (c.2752insA) in exon 26 (figure 1*c*). This mutation causes a frameshift that first results in the introduction of 23 new amino acids after the amino acid at position 917 (p.Q918fsX941) and then results in a truncated form of myosin VI. A fourth mutation was detected in patient III-1 of the SR-107 family (figure 1*d*). This missense mutation was a G to A nucleotide change at position 614 (c.614 G > A), which changes an Arg residue to a Gln at amino acid position 205 (p.R205Q) (figure 1*d*). The arginine at position 205 is conserved among myosins from many different species [17]. The previously solved X-ray structure of *Dictyostelium* myosin II suggested that Arg238 (corresponding to Arg205 of human myosin VI) in the switch I region [18] forms a salt bridge with Glu459 in the switch II region [19]. Therefore, it is anticipated that the p.R205Q mutation in human myosin VI disrupts this salt bridge, which will hamper motor activity. To predict whether this non-synonymous amino acid variant is likely to have a deleterious effect on the phenotype, two prediction programs, PolyPhen2 and SIFT, were applied. The results of both programs indicated that the variation would have a deleterious effect, with a PolyPhen2 score of 1.000 and a SIFT score of 0.00.

**Table 1.** Mutations in the *MYO6* gene (n.a., not available).

exon	nucleotide change	amino acid change	time of onset	phenotype	shape of audiogram
8	c.614G > A	p.R205Q	first decade	progressive, mild to moderate	U-shaped or flat
24	c.2473C > T	p.R825X	n.a.	progressive, moderate to profound	down-sloping
26	c.2752insA	p.Q918fsX941	fifth decade	progressive, moderate	flat
28	c.2971C > T	p.R991X	second decade	progressive, mild to moderate	down-sloping

In the *MYO1A* gene, we identified one variant with a G to A substitution at nucleotide position 1630 (c.1630 G > A) in patient FTM-01. This variant converts a highly conserved Arg to a Trp at amino acid position 544 (p.R544W; figure 1*e*). The results of both prediction programs showed that the variation would have a deleterious effect, with a PolyPhen2 score of 0.998 and a SIFT score of 0.00. None of the five aforementioned variants identified in this study were detected in any of the hundred normal-hearing Korean participants who were examined as controls.

**Figure 2.**
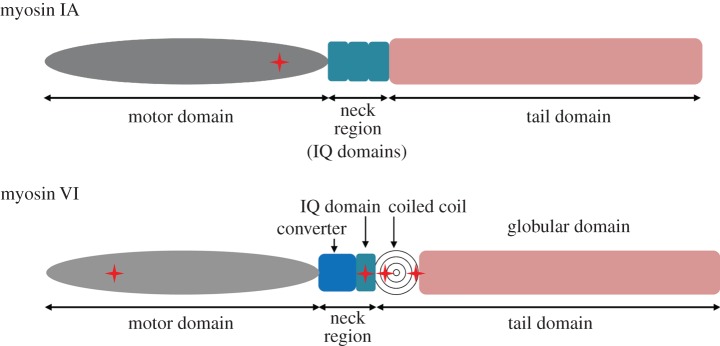
Diagram structure of myosin IA and myosin VI. Schematic of myosin IA and VI show the location of the mutations (cross shape).

Overall, we found one and four mutations in *MYO1A* and *MYO6* in this study, respectively. Figure 2 shows the locations of these mutations.

### 3.2. Protein structure of mutant myosin VI and IA

**Figure 3.**
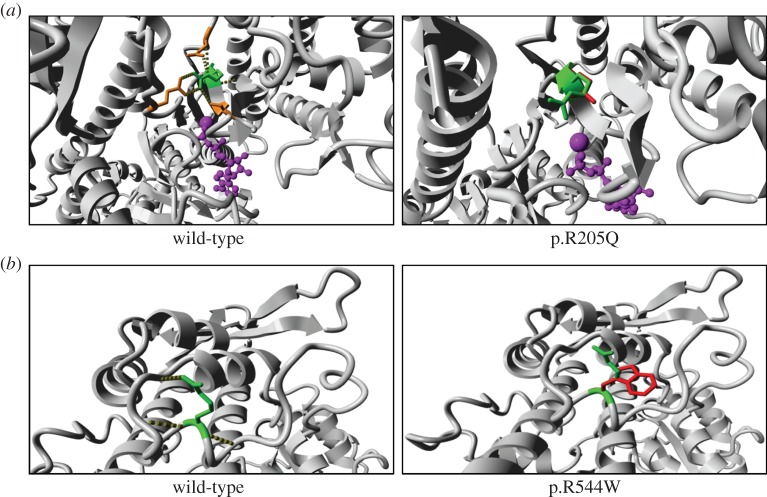
Protein structures of missense mutations in myosin VI and myosin Ia. These images show protein modelling of (*a*) WT and p.R205Q of myosin VI as well as (*b*) WT and p.R544W in myosin IA. These images show a close-up of the mutation site. (*a*) Green, arginine; red, glutamine; magenta, ATP; orange, glutamic acid and serine. (*b*) Green, arginine; red, tryptophan.

We inspected the *MYO6* homology model to determine the structural differences between the wild-type and mutant types, p.R205Q. Arg205 has hydrogen bonds and ionic interactions with several surrounding residues, but the p.R205Q mutation loses all of the ionic interactions because Gln is an uncharged residue (figure 3*a*). Because of the smaller size of Gln, most of the hydrogen bonds are also likely to be lost. In addition, we inspected the *MYO1A* homology model to determine the possible molecular effects of the p.R544W variant. The Arg544 residue in the motor domain of myosin 1A is hydrophilic and forms hydrogen bonds with several surrounding residues (figure 3*b*). The larger and hydrophobic side chain that is introduced by p.R544W cannot form these hydrogen bonds and thus destabilizes the motor domain.

### 3.3. Actin-activated ATPase activity and *in vitro* motility activity of missense mutation in *MYO6*

**Figure 4.**
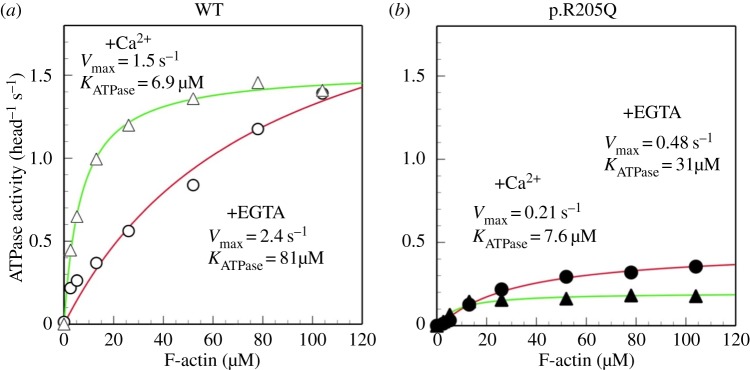
ATPase activity of missense variant (p.R205Q) of myosin VI. This image is F-actin-activated ATPase activity of WT and p.R205Q. Triangles, reaction buffer with 0.1 mM CaCl_2_; circles, reaction buffer with 1 mM EGTA; open symbols, ATPase activity of WT; solid symbols, ATPase activity of (*a*) WT and (*b*) p.R205Q.

**Figure 5.**
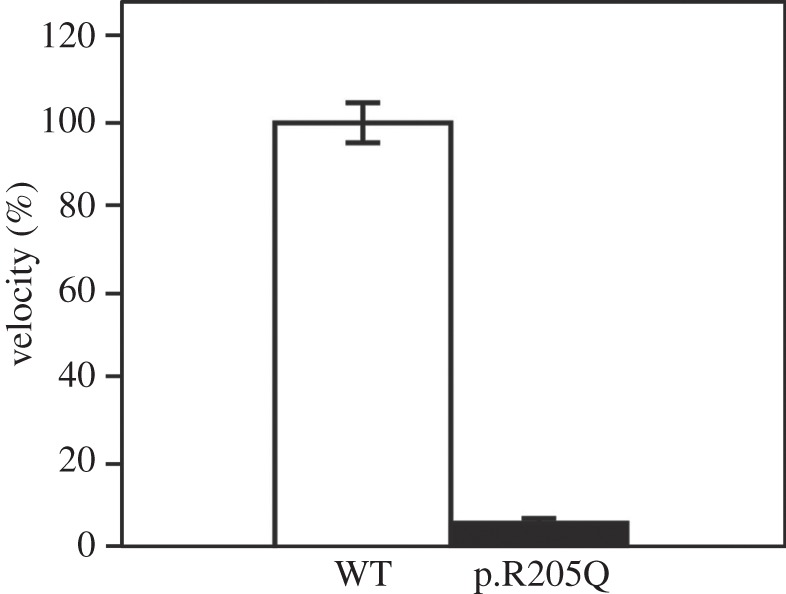
Multi-molecule *in vitro* motility activity of wild-type and p.R205Q mutant of myosin VI. This image shows *in vitro* motility activity of WT (open bar) and p.R205Q (closed bar). Error bars show mean ± s.e. (*n* = 6).

We examined the effect of the p.R205Q mutation on the ATP hydrolysis-associated motor activity of full-length human myosin VI (M6^Full^). The p.R205Q myosin VI hydrolysed ATP in the presence of Mg^2+^ at a constant rate similar to that of the wild-type in the absence of actin. The results suggest that the p.R205Q mutation abolishes neither ATP binding nor hydrolysis. The actin-activated ATPase activity of the wild type (M6^Full^ WT) in 0.1 mM CaCl_2_ showed a K_ATPase_ value that was similar to the tail-truncated mouse myosin VI (M6S1) previously reported [20], and the *V*_max_ was approximately half of the *V*_max_ of M6S1. However, the K_ATPase_ of M6^Full^ WT in ethylene glycol tetraacetic acid (EGTA) was more than 10-fold higher than that in 0.1 mM CaCl_2_ and that of M6S1 in EGTA [20]. These results suggest that the tail portion of the molecule interferes with the interaction with actin in the presence of EGTA. The p.R205Q mutation, however, significantly diminished the actin-activated ATP hydrolysis cycle, which is coupled to the mechanical function of myosin VI. The major effect of the mutation was the significant decrease in *V*_max_ rather than a change in K_ATPase_ (figure 4). In addition, we tested *in vitro* motility activity of M6^Full^ WT and M6^Full^ R205Q myosin VI full length to study interaction of F-actin and myosin. As a result, the velocity of rhodamine-phalloidin labelled F-actin movement was significantly decreased by p.R205Q mutation (figure 5). These results indicate that p.R205Q mutation causes a decrease in the actin-activated ATPase activity, and hampers F-actin movement.

## 4. Discussion

Several studies have reported that mutations in unconventional myosin proteins cause HL in the Caucasian population [6–13]. HL caused by a mutation in the *MYO6* gene was first reported in Snell's waltzer mouse. This strain carries a spontaneous recessive mutation that results in protein deficiency by frameshift mutation (130 bp deletion) [15]. Since then, seven different mutations in the *MYO6* gene have been reported to be associated with DFNA22 deafness in Caucasian families: three missense mutations (p.H246R, p.C442Y and p.R1204W), a nonsense mutation (p.R849X), two splicing mutations (c.897G > T and c.2416 + 2321T > G) and an overexpression mutation [10,21–25]. In addition, three unrelated Pakistani families exhibited homozygosity for three different mutations associated with DFNB37 deafness (c.36insT, p.E216V and p.R1166X) [12]. A number of studies have revealed *MYO6* mutations in the Caucasian and Pakistani populations; however, mutations have not previously been reported in East Asian populations. Our study of Korean patients with ADNSHL identified four novel mutations in the *MYO6* gene that appear to be causally related to deafness. These mutations were neither observed in normal-hearing Korean controls nor in the 1000 Genomes Database and are thus far unique to the Korean deaf population.

Myosin VI is expressed in the early stage of hair cell differentiation [26]. It is located in the hair cell body, the cuticular plate, the pericuticular necklace of hair cells, and between actin and the membrane of stereocilia of hair cells of the organ of Corti [5,27]. The myosin VI dimer can move with a cargo molecule towards the minus end of an actin filament [28]. Moreover, it has been suggested that myosin VI plays an important role in the generation and maintenance of hair cell stereocilia [29], which has been shown in several *in vivo* studies using myosin VI null mice with stereocilia fusion and growth of giant hair cell stereocilia in the cochlea [15,16].

Myosin VI has three functional domains: an N-terminal motor domain or head domain, a C-terminal tail domain and a so-called neck region. The N-terminal motor domain has ATPase activity and binds to actin molecules, whereas the tail domain contributes to the dimerization of myosin molecules and cargo binding. The neck region is located between the head and tail domains, and contains light chain- or calmodulin-binding IQ motifs [3,30,31]. Myosin VI is a motor protein that uses energy derived from ATP hydrolysis by actin-activated ATPase [31], and half of all myosin VI mutations have been found in the motor domain [10,12,21,23]. Because Snell's waltzer mouse with an alternatively spliced, truncated myosin VI protein has shown disturbing dimerization [15,23], we expect that the two nonsense mutations and the frameshift mutation disrupt dimerization in a similar manner, resulting in a myosin VI null phenotype. In addition, the p.R238E mutation of Dictyostelium myosin II [32–34] and the p.R247A/p.R247E mutation of smooth muscle myosin II [35] were previously reported to abolish actin-activated ATPase activity. The present functional studies revealed that mutation of the conserved Arg205 to Gln does not abolish the actin activity of myosin VI but significantly decreases *V*_max_. Because the Gln side chain contains the –NH_2_ group, it is possible that it can still form a hydrogen bond with the Glu side chain in switch II, but we cannot exclude the possibility that the active site pockets are slightly different between myosin II and myosin VI. Taken together, these findings indicate that the p.R205Q mutation causes a myosin VI null function via reduced ATPase activity in the motor domain.

Myosin IA is one of the most abundant components of the enterocyte brush border, and plays critical roles in the interface between the membrane and actin cytoskeleton [36]. The p.E385D missense mutation is considered causative largely because it was demonstrated to disrupt actin movement by lowering ATPase activity [37]. We observed one variant, p.R544W, in the *MYO1A* gene. This Arg544 residue is located in a conserved region of the myosin IA motor domain in vertebrates. Our *in silico* analyses suggest that p.R544W may be a disease-causing variant, because this mutation causes destabilization of the motor domain because hydrogen bonds cannot be formed. However, further *in vivo* or *in vitro* analyses are required to determine whether this variant is truly causative or whether it is a rare polymorphism.

In conclusion, we identified five novel mutations in *MYO1A* and *MYO6*, and analysed their characteristics by *in vitro* and *in silico* analyses. We explained in detail that the p.R205Q mutation in myosin VI decreases ATPase activity significantly.

## 5. Material and methods

### 5.1. Subjects and clinical evaluations

We selected 53 unrelated Korean patients with non-syndromic HL whose families exhibited an autosomal dominant inheritance pattern. All subjects were recruited from the Yonsei University Hospital, Seoul, Korea, the Soree Ear Clinic, Seoul, Korea or the Patima Hospital, Daegu, Korea. Following a physical and otoscopic examination, pure tone audiometry (PTA) was performed in a sound-attenuated room. We determined the average thresholds for a frequency range of 250–8000 Hz. One hundred unrelated Koreans diagnosed as normal by PTA test were recruited as control subjects.

### 5.2. Genetic analysis

Genomic DNA was extracted from peripheral blood using a FlexiGene DNA extraction kit (Qiagen, Hilden, Germany) or from buccal cells using a Puregene Buccal Cell Core kit (Qiagen). To analyse the *MYO1A* and *MYO6* gene sequences, all of the exons and exon–intron boundary regions in each gene were amplified by polymerase chain reaction (PCR) using specifically designed primer pairs. We performed cycling PCR using an ABI Big Dye Terminator v. 3.1 Cycle Sequencing kit (Applied Biosystems, Foster City, CA). PCR products were sequenced using an ABI 3130XL genetic analyser (Applied Biosystems). The sequence data were analysed using Chromas Pro (v. 1.5) software (Technelysium, Pty Ltd., Tewantin, Queensland, Australia) and Seqscape (v. 2.5) software (Applied Biosystems), and the sequences were compared with the corresponding sequences in GenBank (accession no. NM_001256041.1 for *MYO1A* and accession no. NM_004999.3 for *MYO6*). The amino acid sequences of various vertebrate species were aligned using the Clustal W2 program (http://www.ebi.ac.uk/Tools/msa/clustalw2/) to evaluate whether amino acid substitutions occur in conserved regions of the proteins. The pathogenic effect of these substitutions was predicted using SIFT (http://sift.jcvi.org/) and PolyPhen2 (http://genetics.bwh.harvard.edu/pph2).

### 5.3. Protein modelling

Because no experimentally solved structures exist for either *MYO1A* or *MOY6*, we built homology models for these proteins. The YASARA and WHAT IF Twinset was used (with default parameters) for model building and subsequent analyses [38]. The model for *MYO1A* was constructed using the PDB file 2dfs [39] as a template. This file contains the structure for myosin V from chicken, which is 37% identical to *MYO1A* over 770 residues. The modelled domain of *MYO1A* contains the location of the newly identified mutation p.R544W. The model for myosin VI was constructed from myosin VI of wild boar, which is 98% identical to the human sequence over 784 residues [40]. The point mutations in *MYO6* were analysed using this model.

### 5.4. Generation of myosin VI constructs and isolation of the proteins

Human myosin VI cDNA clones containing nucleotides 1–1285 were obtained from a human kidney cDNA library and inserted into the pFastbac1 (Invitrogen, Carlsbad, CA) baculovirus transfer vector at the polylinker region. For wild-type recombinant myosin VI (M6^Full^WT), a c-myc and FLAG tag sequence (ACGCGTGAGCAAAAGCTCATTTCTGAAGAGGACTTGTCGCGTGATTATAAAGATGATGATGATAAA) was introduced between the 3′ end of the myosin VI cDNA and the stop codon to aid purification of the recombinant protein. The p.R205Q mutation of myosin VI was generated by PCR-based site-directed mutagenesis [41] using Pfu DNA polymerase. The presence of the mutation was confirmed by sequencing. Expression and purification of the recombinant myosin VI was performed as described previously [20].

### 5.5. ATPase assay

ATPase activity was measured at 25°C in a buffer that contained 30 mM KCl, 20 mM MOPS-KOH (pH 7.5), 3 mM MgCl_2_, 2 mM ATP, 5 µM CaM, 40 unit ml^−1^ pyruvate kinase, 2.5 mM phosphoenolpyruvate, 1 mM EGTA or 0.2 mM CaCl_2_, 11–14 µg ml^−1^ M6^Full^ WT or the mutant, and various concentrations of F-actin ranging from 0 to 104 µM. The liberated pyruvate was determined as described previously [42]. To calculate the ATPase activity, protein concentrations of M6^Full^ WT and the mutant were determined using Coomassie Plus Assay Reagent (Thermo Scientific, Waltham, MA). The molecular weight of M6^Full^ WT was assumed to be 185 kDa (151.4 kDa M6 heavy chain and 2 × 16.8 kDa calmodulin light chains).

### 5.6. *In vitro* motility assay

*In vitro* motility of Rhodamine-labelled F-actin was measured using a cooled-CCD camera (Sensi Cam^QE^, Cooke Co.) equipped with an Olympus IX-51-based fluorescence microscope [20]. The assay was carried out in a buffer containing 25 mM KCl, 20 mM MOPS-KOH (pH7.5), 5 mM MgCl_2_, 1 mM EGTA, 2 mM ATP, 216 µg ml^−1^ glucose oxidase, 36 µg ml^−1^ catalase, 4.5 mg ml^−1^ glucose, 10 mM dithiothreitol, 5 µM calmodulin and 15 nM F-actin at 21°C. Each of wild-type and the mutant M6^Full^ with the appropriate concentration was attached to the same coverslip separated with double-adhesive tape, and F-actin movements were captured at every 2 (wild-type) or 8 (p.R205Q) seconds. The velocity was calculated from the distance and the elapsed time in the video images.

## Notes

This study was approved by the Institutional Review Board of the Yonsei University College of Medicine, and written informed consent was obtained from all participants.
